# The influence of moonlight and lunar periodicity on the efficacy of CDC light trap in sampling *Phlebotomus (Larroussius) orientalis* Parrot, 1936 and other *Phlebotomus* sandflies (Diptera: Psychodidae) in Ethiopia

**DOI:** 10.1186/s13071-015-0723-7

**Published:** 2015-02-15

**Authors:** Araya Gebresilassie, Solomon Yared, Essayas Aklilu, Oscar David Kirstein, Aviad Moncaz, Habte Tekie, Meshesha Balkew, Alon Warburg, Asrat Hailu, Teshome Gebre-Michael

**Affiliations:** Department of Zoological Sciences, Addis Ababa University, Addis Ababa, Ethiopia; Department of Biology, College of Natural Science, Jigjiga University, Jigjiga, Ethiopia; Department of Microbiology and Molecular Genetics, The Institute of Medical Research Israel-Canada The Kuvin Center for the Study of Infectious and Tropical Diseases, Faculty of Medicine, The Hebrew University, Hadassah Medical School, Jerusalem, Israel; Aklilu Lemma Institute of Pathobiology, Addis Ababa University, Addis Ababa, Ethiopia; Department of Microbiology, Immunology and Parasitology, School of Medicine, Addis Ababa University, Addis Ababa, Ethiopia

**Keywords:** CDC light traps, Lunar cycles, *Phlebotomus orientalis*, Visceral leishmaniasis

## Abstract

**Background:**

*Phlebotomus orientalis* is the main sandfly vector of visceral leishmaniasis in the north and northwest of Ethiopia. CDC light traps and sticky traps are commonly used for monitoring sandfly populations. However, their trapping efficiency is greatly influenced by various environmental factors including moonlight and lunar periodicity. In view of that, the current study assessed the effect of moonlight and lunar periodicity on the performance of light traps in collecting *P. orientalis*.

**Methods:**

Trapping of *P. orientalis* and other *Phlebotomus* spp. was conducted for 7 months between December 2012 and June 2013 using CDC light traps and sticky traps from peri-domestic and agricultural fields. Throughout the trapping periods, collections of sandfly specimens were carried out for 4 nights per month, totaling 28 trapping nights that coincided with the four lunar phases (viz., first quarter, third quarter, new and full moon) distributed in each month.

**Results:**

In total, 13,533 sandflies of eight *Phlebotomus* species (*P. orientalis*, *P. bergeroti*, *P. rodhaini*, *P. duboscqi, P. papatasi*, *P. martini*, *P. lesleyae* and *P. heischi*) were recorded. The predominant species was *P. orientalis* in both trapping sites and by both methods of collection in all lunar phases. A significant difference (*P* < 0.05) was observed in the mean numbers of *P. orientalis* and other *Phlebotomus* spp. caught by CDC light traps among the four lunar phases. The highest mean number (231.13 ± 36.27 flies/trap/night) of *P. orientalis* was collected during the new moon phases, when the moonlight is absent. Fewer sandflies were attracted to light traps during a full moon. However, the number of *P. orientalis* and the other *Phlebotomus* spp. from sticky traps did not differ in their density among the four lunar phases (*P* = 0.122).

**Conclusion:**

Results of the current study demonstrated that the attraction and trapping efficiency of CDC light traps is largely influenced by the presence moonlight, especially during a full moon. Therefore, sampling of sandflies using light traps to estimate population density and other epidemiological studies in the field should take the effect of moonlight and lunar periodicity into account on the trapping efficacy of light traps.

## Background

Phlebotomine sandflies are small, fragile, nocturnally active nematoceran insects with weak flight capabilities. In the Old World, females of the genus *Phlebotomus* Rondani & Berté, 1840 have considerable public health importance as vectors of the leishmaniases, and sandfly fever viruses [1,2]. In addition, sandfly bites cause allergic reactions and substantial irritation in sensitive people.

Visceral leishmaniasis (VL) also known as Kala-azar, caused by infection with *Leishmania donovani* complex is transmitted by the sandfly *P. orientalis* in Sudan, South Sudan, northern and south western Ethiopia [3-5]. This species is frequently associated with *Acacia-Balanite*s-*Ziziphus* woodlands and vertisols (black cotton soils) [6,7].

In order to understand sandfly bionomics, it is imperative to sample sandflies in their different habitats. Commonly used techniques for monitoring sandfly populations are CDC light traps and sticky traps [8-10]. However, their trapping efficiency is greatly influenced by various environmental factors such as weather (wind speed, temperature, rainfall, relative humidity, night-length) and lunar illumination [11-13]. The lunar phase is known to influence adult flight behavior of many insects including those of the order Diptera, particularly Culicidae [14,15]. Moonlight variations could also directly influence mosquito activity [16]. However, another study found no direct influence of the lunar cycle on Brazilian populations of mosquitoes [17]. Results on the effect of lunar phases on sandflies activity are contradictory. In Brazil [18], it was indicated that fewer sandflies were attracted to light traps during full moon. Similarly, sandfly species of *Lutzomyia intermedia, Lu. migonei*, and *Lu. fischeri* had higher abundance in the light traps during the new and half moon phases [19]. However, a recent study in Italy [12] reported that *P. perniciosus* and *Sergentomyia minuta* were mainly collected during the full moon phases, while no significant differences in the capturing of sandflies was observed among lunar phases in Kenya [20].

In Ethiopia, it has frequently been observed that CDC light trap catches of sandflies (both *Phlebotomus* spp. and *Sergentomyia* spp.) were almost empty during full or partial moonlit nights, although they have never been properly evaluated (Gebre-Michael and Balkew, unpublished data). As a result, field visits for sampling of sandflies have always been planned according to the phases of the moon when moonlight is completely absent during the whole or for most of the night. Therefore, the present investigation was carried out to elucidate quantitatively the effect of moonlight and lunar periodicity on the performance of light traps in collecting *P. orientalis* during the active periods in northern Ethiopia where VL is becoming an emerging disease.

## Methods

### Study area

Entomological investigation was conducted in Geza Adura in one of the rural village of Tahtay Adiyabo district (14°22’27”N/ 37°44’36”E) in Tigray Regional State, Northern Ethiopia. The administrative center of the district is located 1,117 km north of Addis Ababa and 402 km north-west of Mekelle, the capital of Tigray Regional State. The area is lowland plains with an average altitude of 1,028 meters above sea level. The climate is generally sub-tropical-arid, with an extended dry period of nine to ten months. The area has a uni-modal pattern of rainfall (July-September) with a mean annual precipitation of about 600 mm. March to May is the hottest part of the year with an average temperature of 39°C at noon and January is the coldest one with an average temperature of 14.2°C at night.

The villages are situated on rocky hills surrounded by large farm fields of vertisols alternating with large tracts of red clay soil. The inhabitants are mainly engaged in the production of cereals and oilseeds and raising domestic animals.

### Sandfly sampling strategies

For sandfly collections, two sampling sites were selected: peri-domestic habitats (compounds of human and animal shelters), and agricultural fields with scattered and mixed trees mainly of *Balanites*-*Ziziphus-Acacia* and some scrub vegetation. Sandfly trapping was conducted for 7 months between December 2012 and June 2013. In the area, *P. orientalis* has been determined to have the greatest overall activity between December and June, reaching its peak density between March and April (Gebresilassie *et al*., unpublished data). Throughout the trapping periods, collections of sandfly specimens were carried out for four nights per month, totaling 28 sampling nights. Sampling nights were categorized into four nights so as to coincide with the four lunar phases (viz., first quarter, third quarter, new and full moon) distributed in each month.

#### CDC light traps

Sandflies were collected using CDC miniature light traps (John W. Hock, Gainesville, FL) (n = 112/28 nights). For this purpose, two CDC light traps/night/lunar phase were deployed in representative sites (inside compounds and animal shelters) of peri-domestic habitats throughout the sampling seasons. Simultaneously, another two light traps were operated in agricultural fields (open cultivated vertisols and between sparsely placed *Acacia* and *Balanites Balanites* trees) in similar way as peri-domestic habitat. In all those sampling periods, the CDC light traps were suspended with the fan 40–50 cm above the ground level in, which were devoid of objects that could potentially shield the exposure of traps to moonlight source.

#### Sticky traps (STs)

A4-sized white sticky traps (n = 280/28 nights) of polypropylene sheets coated with sesame oil were used for capturing sandflies from all sampling habitats. The five sticky traps were randomly installed horizontally: on cracked walls (2 STs), a stone pile produced by a collapsing hut (1 ST), and animal enclosures (2 STs) in the peri-domestic environment. At the same time, another five sticky traps were placed horizontally: over cracked vertices (2), dry riverbed (1), branches of scrub vegetation (1) and loose stone walls surrounding farm yards (1). Traps were set up for four nights every month divided among the four lunar phases in each habitat.

Both CDC light traps and sticky traps were deployed in the two collection sites 1 h before sunset and collected at dawn the next morning. Then, traps containing sandflies were transported to the field laboratory, where sandflies were sorted by sex and genus (*Phlebotomus* or *Sergentomyia* spp.). The remaining specimens were preserved in 70% ethanol in labeled vials for later processing and identification to species level.

### Sandfly identification

Collected sandflies were dissected and mounted on microscope slides in Hoyer’s medium with their heads separate from thoraces and abdomens. Species were identified based on the morphology of the external genitalia of males and the pharynx, antennal features and spermathecae of females, using different keys, [21,22] and other publication [23].

### Data on moon phases and percent illumination

Timings of moonrise and moonset, tables of moon-phases, and the percent illumination of the moon corresponding to each night of moon phase were downloaded from the Astronomical Applications Department of the US Naval Observatory (http://www.vercalendario.info/en/moon/ethiopia-2013.html) and Astronomy Know How Moon Percentage Illumination (http://www.astronomyknowhow.com/month-percentage.php) and was adjusted to Standard Time.

### Data analysis

The sandfly trap-yields captured in different lunar phases and habitats by light traps and sticky traps were log-transformed [log (n + 1)] to fit normal distribution and tested for normality by 1-Sample Kolmogorov-Smirnov Z test (K-S). Thereafter, one-way Analysis of Variance (ANOVA) was used to compare the mean number of *P. orientalis* using CDC light traps during the four lunar phases. Similarly, the mean numbers of other *Phlebotomus* spp. captured in CDC light traps and on sticky traps were analyzed using one-way ANOVA. Tukey’s Studentized test post hoc analysis was utilized for mean separation where ANOVA was significant. Linear correlation analysis was also applied to determine the relationship between mean number of *P. orientalis*/light trap/night and the percentage of moonlight available for the corresponding day. Otherwise, the non-parametric equivalent tests of Kruskal-Wallis and Mann–Whitney-*U* were used when trapping data did not conform to the normal distribution. Kruskal-Wallis test was used to compare the mean number of *P. orientalis* caught on sticky traps among the four lunar phases. The Mann–Whitney *U*-test was also followed to compare the mean numbers of sandfly specimens captured per trap. Statistical tests were considered significant if *P* < 0.05. All statistical analyses were carried out using IBM SPSS statistics, version 19 for Windows (SPSS Inc., Chicago, IL, USA) and Microsoft® Office Excel 2007. Though log-transformed values were used for the analyses, actual values are reported in the text, figures and tables.

## Results

### Sandfly species composition

**Figure 1:**
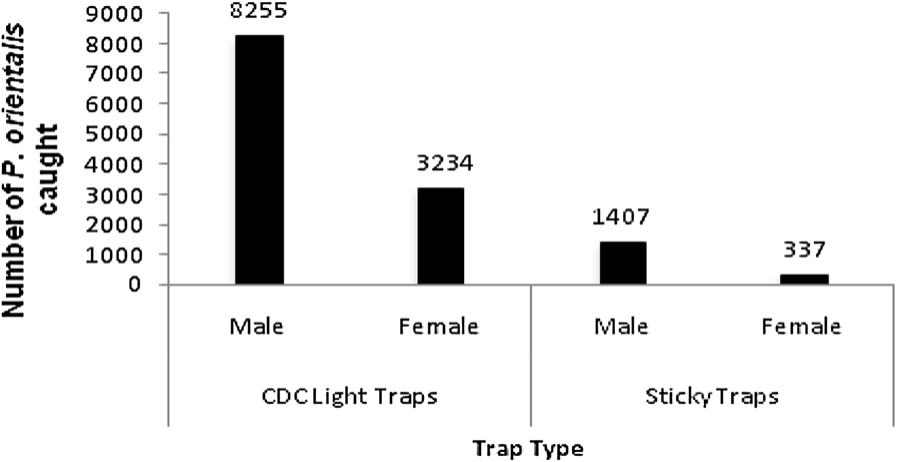
***Phlebotomus orientalis*** **male and female sandflies caught by CDC light traps and sticky traps in peri-domestic and agricultural fields in Tahtay Adiyabo district, December 2012-June 2013.**

In total, 13,533 sandfly specimens belonging to eight species of the genus *Phlebotomus* were collected: 11,667 in light traps and 1,866 on sticky traps (Table 1). The species comprised *P. orientalis*, *P. bergeroti*, *P. rodhaini*, *P. duboscqi, P. papatasi*, *P. martini*, *P. lesleyae* and *P. heischi*. The most abundantly collected species was *P. orientalis* (97.78%) followed by *P. bergeroti* (0.75%). The other species constituted less than 1.5% of the total collection. The mean number of *P. orientalis* captured in light traps was higher than in sticky traps in paired collections (light trap vs. sticky trap); with mean ± SE of 122.06 ± 15.36 vs.6.23 ± 1.24 in light traps vs. sticky traps (Mann Whitney *U*-test, *P <* 0.05). Additionally, the total number of *P. orientalis* males caught by both light traps and sticky traps in the two collection sites was higher than that of females (9,663 males: 3571 females). The male/female sex ratio for *P. orientalis* was 2.55 and 4.18 for light traps and sticky traps, respectively (Figure 1).

**Table 1:** ***Phlebotomus*** **species captured using CDC light traps and sticky traps in peri-domestic and agricultural fields in Tahtay Adiyabo district, December 2012-June 2013**

	**Sampling habitats**					
**Sandfly species**	**Peri-domestic**		**Agricultural field**			
	**CDC traps**	**Sticky traps**	**CDC traps**	**Sticky traps**	**Total**	**Relative frequency (%)**
*Phlebotomus orientalis*	5,943	1,175	5,546	569	13,233	97.78
*P. bergeroti*	72	9	11	9	101	0.75
*P. rodhaini*	9	8	41	8	66	0.49
*P. duboscqi*	7	1	3	0	11	0.08
*P. papatasi*	3	2	2	2	9	0.07
*P. martini*	0	0	3	0	3	0.02
*P. lesleyae*	11	21	9	57	98	0.72
*P. heischi*	2	2	5	3	12	0.09
Total	6,047	1,218	5,620	648	13,533	100

### Abundance of sandfly in peri-domestic habitat and agricultural fields

Overall, 7265 and 6268 sandfly species were collected in peri-domestic and agricultural fields on the periphery of the settlements, respectively (Table 1). There was no significant difference in the number of *P. orientalis* observed between the two habitats (Mann Whitney *U*-test, *P* > 0.05), though a large number of *P. orientalis* were collected in peri-domestic habitat. Higher numbers of *P. bergeroti* were also collected in peri-domestic than agricultural fields. *P. rodhaini* and *P. lesleyae*, which are the next most abundant species, were captured more in agricultural fields, but were less common than *P. orientalis*.

### Effect of lunar phases on the trap-yield for capturing *P. orientalis*

**Figure 2:**
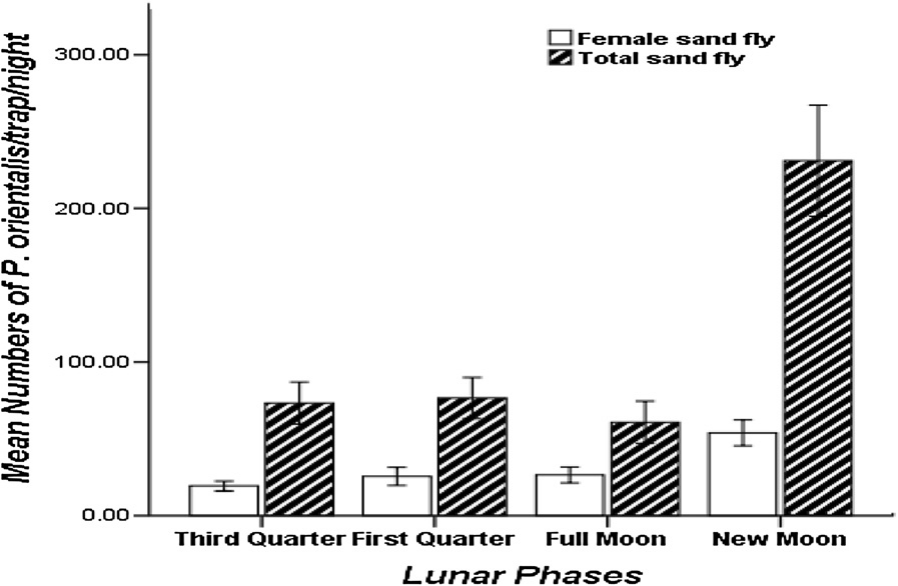
**Mean numbers (±SE) of total and female** ***P*** **.** ***orientalis*** **/trap/night captured during different lunar phases with CDC light traps from peri-domestic and agricultural fields at Tahtay Adiyabo district, December 2012-June 2013.**

The analysis of the data of CDC light trap catches indicated a highly significant difference in the attraction response of *P. orientalis* in different lunar phases (ANOVA, F _(df=3)_ = 13.96; *P* < 0.05, Figure 2). The abundance of *P. orientalis* was significantly higher during the new moon phase with a mean of 231.13 ± 36.27 flies/trap/night. The mean number of *P. orientalis* (60.64 ± 13.72 flies/trap/night) collected in light traps on moonlit nights was around 25% of the catch during a non-moon phase. There was no significant density difference among the first quarter, third quarter and full moon phases (*P* > 0.05) (Figure 2).

There was a significant difference between the mean numbers of *P. orientalis* females captured in the four lunar phases (ANOVA, F _(df=3)_ = 4.86, *P* < 0.05; Figure 2). The mean number of *P. orientalis* females captured during new moon phases was higher than other lunar cycles. In particular, the mean number of female *P. orientalis* was substantially reduced during the moonlit nights around the full moon (Figure 2).

**Figure 3:**
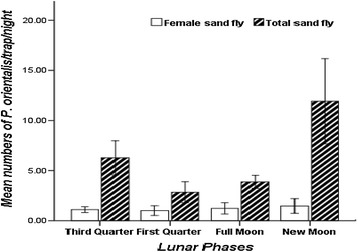
**Mean numbers (±SE) of total and female** ***P*** **.** ***orientalis*** **/sticky trap/night captured in different lunar phases from peri-domestic and agricultural fields in Tahtay Adiyabo district, December 2012-June 2013.**

Contrary to CDC light traps, different lunar phases had no significant effect on the mean numbers of *P. orientalis* intercepted by sticky traps (Kruskal-Wallis test, *P* > 0.05, Figure 3). The mean numbers of *P. orientalis* captured during new, third quarter, first quarter, and full moon phases were: 11.0 ± 4.25, 6.27 ± 1.7, 2.85 ± 1.04, and 3.87 ± 0.65/trap/night, respectively. Likewise, non-significant differences were observed in the mean numbers/trap/night of female *P. orientalis* intercepted by sticky traps during the four lunar cycles. The mean density of *P. orientalis* females caught during the four lunar phases ranged from 1.01 to 1.47/trap/night (Figure 3).

### Effect of lunar phases on the trap-yield for capturing other *Phlebotomus* spp

**Figure 4:**
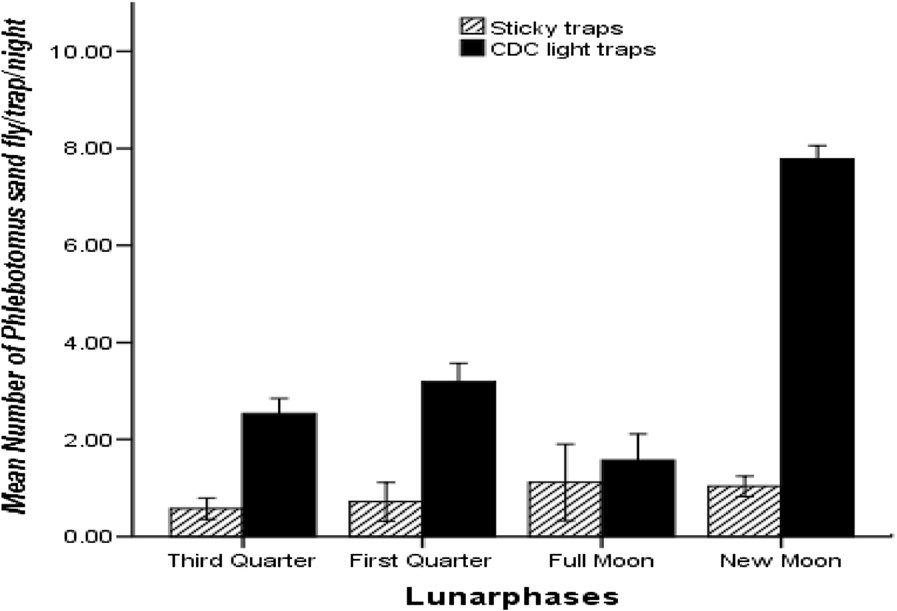
**The mean (±SE) number of pooled** ***Phlebotomus*** **species caught in Tahtay Adiyabo district during four lunar phases per trap per night in Tahtay Adiyabo district, December 2012-June 2013.**

The effect of moonlight on catches of other *Phlebotomus* spp. (i.e., *P. bergeroti*, *P. rodhaini*, *P. duboscqi, P. papatasi*, *P. martini*, *P. lesleyae* and *P. heischi*) pooled was also analysed since catches of each species was low in density. Thus, the four lunar phases had a significant effect on the mean numbers of the pooled *Phlebotomus* spp., which were captured by CDC light traps (ANOVA, F _(df=3)_ = 50.19; *P* < 0.05, Figure 4). Nearly twice the mean number of *Phlebotomus* species/trap/night was found during a new moon phase than the other three phases combined. Nonetheless, the difference between the total numbers of sandflies collected using sticky traps during the four lunar phases was not significant for *Phlebotomus* species (ANOVA, F _(*df=3*)_ = 0.305; *P* >0.05). Mean numbers of *Phlebotomus* sandfly specimens captured on sticky traps during the four lunar phases were small, which ranged from 0.56 for new moon phase to 2.07/trap/night for full moon phase.

### Relationship between moonlight and light trap catches

**Figure 5:**
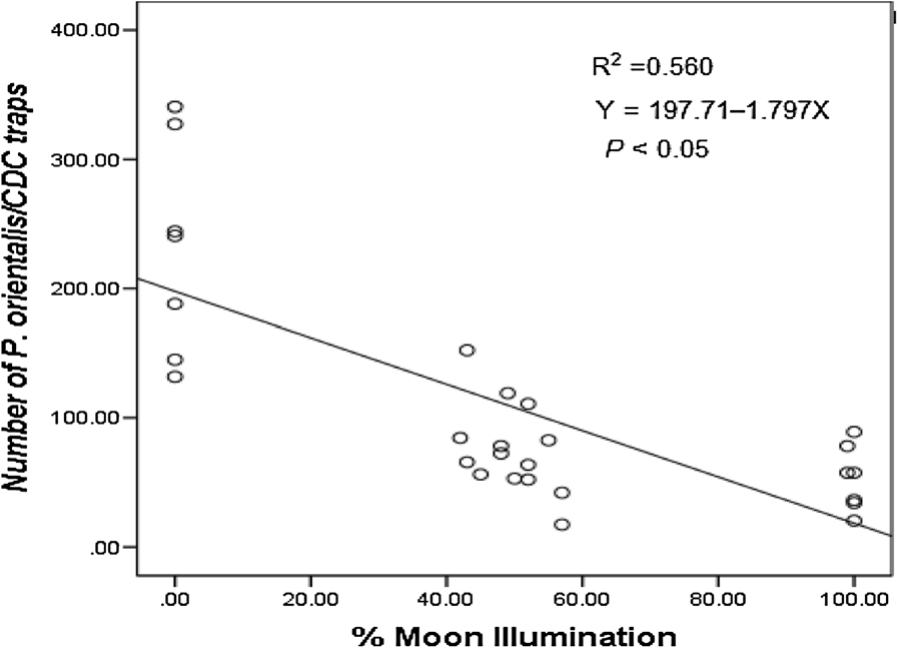
**A linear decreases in the number of** ***P. orientalis*** **collected/light trap/night with the increase in the percentage of moonlight.**

Regression analysis revealed a highly significant inverse linear relationship between the percentage of moonlight illumination and light trap catches of *P. orientalis* (R^2^ = 0.560, df = 27, *P* <0.05) (Figure 5). The number of *P. orientalis* collected by CDC light traps decreased linearly as the percentage of moon illumination increased.

## Discussion

The current study assessed the possible influence of moonlight and lunar periodicity on the efficacy of CDC light traps for sampling *P. orientalis* and other *Phlebotomus* species in northern Ethiopia during different moon phases. During 28 trapping nights, 13,533 sandflies of *Phlebotomus* spp. were collected using CDC light traps and sticky traps in peri-domestic and agricultural habitats, respectively. In this collection, eight species of the genus *Phlebotomus* and several *Sergentomyia* spp. were trapped, not considered in this report. The species recorded in the current investigation are in agreement with previous documentations on the species composition of the genus of *Phlebotomus* in the area [24].

The results of the present study, which is the first of its kind in Ethiopia, clearly demonstrated the significant effects of lunar phases and fractionation of moon illumination on the efficacy of CDC light traps for sampling *P. orientalis* while the effect on sticky trap collections was insignificant. As fullness of the moon increased, the attraction response of *P. orientalis* to light traps significantly decreased. Moreover, the mean number of *P. orientalis* females caught was twice as high during phases with no moon than with a full moon. Prominently, regression analysis ascertained that the intensity of moon illumination had the strongest influence on the mean density of *P. orientalis* caught by CDC light traps. The steeper slope in the figure revealed that with increased percentage of moon surface illumination there would be increased ambient light leading to decreased numbers of sandflies collected (Figure 5). The results clearly imply that lunar phases and illuminations have an adverse effect on the trapping efficiency of light traps for sampling disease vectors in the field.

As phototrophic insects, sandflies exhibit positive phototaxis and are, therefore, attracted to light traps [25,26], which may be adversely affected by increased intensity of moon illumination [27]. Santos-de Marco *et al*. found that the attractiveness of light traps toward *Lu. intermedia* was decreased during the brightest (gibbous and full moon) phases of the moon than the dark phases (new and crescent) [28]. Studies in Brazil [29] and in Iraq [11] also reported similar significant negative correlation between moonlight intensity and number of sandflies collected in CDC light traps as shown in the present observation on *P. orientalis* and other *Phlebotomus* spp.

Contrary to the present findings in our work and the above mentioned reports, other investigators reported different results on the role of lunar cycles on the trapping performance of various light traps. Light trapping in Colombia [30] resulted in increased abundance of *Lu. longipalpis* in moon nights as compared to dark nights. A recent study in Italy [12] also indicated that *P. perniciosus* and *S. minuta* were mainly collected during the full moon phases. However, no differences in the number of *Phlebotomus* spp. and *Sergentomyia* spp. caught in CDC light traps were observed among lunar phases in Kenya [20]. Such variations of observations could partly be explained by the variation in the response of sandfly species to light sources in the lunar phases and the experimental procedures followed by different investigators [29].

Decreased flight activity and diminishing of collecting distance as a cause for a drop in the efficiency of light trappings due to moonlight are proposed [31]. For increased moon illumination in the environment, there is increased ambient light that could compete with the light from the trap, thereby reducing the number of sandflies that will pick up the visual cue from the light trap and be attracted to it [11]. Similarly, the observation that ambient moonlight competes with light traps is supported by the effect of cloud cover on the number of individual noctuid moths caught [32].

In other sampling techniques such as sticky traps and landing/biting catches that do not rely on a light source, the possible impact of lunar illumination on the trapping efficacy and sandfly activity might be minimal. For example, *P. papatasi* collection in Egypt using sticky traps was not significantly affected by lunar phases [33], which is comparable with our observations on sticky traps collections.

## Conclusions

Results of the current study indicated that during the full moon, the trapping efficiency of light traps was minimal, but as the fraction of moon illumination decreased, the mean number of sandflies caught increased with peak around the new moon. In contrast, the total number of *P. orientalis* collected in sticky traps appeared to be unaffected by the lunar cycles. Therefore, it would seem that the lunar phase is a factor that should be taken into account when planning or analyzing data on sandfly captures in light traps.
